# A real-life study of juvenile idiopathic arthritis from two Brazilian referral centers for pediatric rheumatology

**DOI:** 10.1186/s42358-025-00459-2

**Published:** 2025-06-11

**Authors:** Ana María Loroño Terrazas, Raúl Gutiérrez Suárez, Reinan Tavares Campos, Claudio Arnaldo Len, Nadia Emi Aikawa, Jade Dib Fernandez, Mayra Lisyer Dantas, Clóvis Artur Silva, Gleice Clemente, Maria Teresa Terreri

**Affiliations:** 1 Paediatric Rheumatology Unit, Paediatrics Department, Universidade Federal de Sao Paulo, Bacelar Street, 173, 12 floor, Sao Paulo, SP 04026-000 Brazil; 2 Shriners Hospital for Children, Mexico City, México; 3 Children’s Institute, Faculdade de Medicina, Hospital das Clínicas HCFMUSP, Universidade de Sao Paulo, Sao Paulo, Brazil

**Keywords:** Juvenile idiopathic arthritis, Remission, Inactive disease, Disease activity, Minimal disease activity, Treatment

## Abstract

**Background:**

Juvenile idiopathic arthritis (JIA) is the most common rheumatic disease in childhood, but its outcomes are still difficult to determine. We aimed to obtain outcome measurements of disease activity, functional capacity, disease damage, and therapeutic response, at one-year follow-up study on a real-life basis.

**Methods:**

An observational JIA cohort from two referral centers for pediatric rheumatology in Brazil Pediatric Rheumatology Centers was carried out over a period of one year. Clinimetric validated outcome measurements were applied over four visits. Multivariable logistic regression was performed to evaluate baseline variables associated with the following outcomes after one year of follow-up: disease activity, Minimal Disease Activity (MDA), disease flare, remission on medication and remission off medication.

**Results:**

A total of 127 patients were included in the study. Eighty-three (65.4%) were females, and median time to diagnosis was 6.0 months. JADAS71 and CHAQ improved during follow-up (mean Vbaseline 7.05 ± 9.3 vs. V52 3.52 ± 8.4; 0 ± 0.5 vs. 0.14 ± 0.4, *p* < 0.001, respectively) as well as JIA-ACR 30, 50, 70 and 90 (Vbaseline 79.8% vs. V52 88.8%; 71.4% vs. 84.1%; 59.7% vs. 77.6%; 49.6% vs. 69.2%, *p* < 0.05, respectively). Inactive disease was present in 33% of patients at baseline and in 67.9% at V52 (*p* < 0.001). Remission on medication, remission off medication and MDA were present in 46%, 11%, and 80.6% of patients at V52, respectively. Extra-articular and articular damage were not common at baseline (0.3 ± 0.7 and 1.1 ± 3.4 respectively) and did not decrease significantly during the period of the study. The presence of active joints 46/101 (45.5%) at baseline reduced the chance of the patient achieving remission off medication at the last visit by 84% (OR = 0.16; CI 95% from 0.03 to 0.91, *p* = 0.039).

**Conclusion:**

This study showed improvement in clinimetric outcome measurements and therapeutic responses in an inception cohort of JIA patients. The presence of active joints at baseline is related to not achieving remission at last visit. Long-term prospective and multicenter studies are needed to better assess the outcome of JIA and the differences among JIA subtypes.

## Background

Juvenile idiopathic arthritis (JIA) is the most frequent autoimmune rheumatic disease in childhood and is characterized by chronic arthritis with different phenotypes [1]. Therapeutic advances have improved significantly and consequently the prognosis has become progressively better [2]. In light of this, therapeutic response criteria and disease activity scores have been developed and improved over the last few decades, and also, definitions of disease inactivity, disease remission, and functional limitation, in order to better define outcomes [3–5].

Inactivity can be difficult to achieve in some patients, especially those with severe or long-term disease. A relatively new concept, Minimal Disease Activity (MDA), has been used for those patients with favorable response, but who have not achieved disease inactivity [6]. Both articular and extra-articular damage from the disease can also be measured through clinimetric definitions [7].

International studies related to epidemiology, clinical features, diagnosis, and follow-up of JIA patients are scarce, particularly those using overall clinimetric evaluations, such as visual analogic scale (VAS), Juvenile Arthritis Disease Activity Score (JADAS) [4], Childhood Health Assessment Questionnaire (CHAQ) [8], Juvenile Arthritis Damage Index (JADI) [7], and JIA-American College of Rheumatology (ACR) response [3], in real life scenarios in Latin American centers [9, 10]. Additionally, none of them evaluated several measures of clinimetric outcomes and therapeutic responses concomitantly in a longitudinal study in pediatric rheumatology centers in a Latin American country.

Therefore, the aims of this study were to obtain outcome measures of disease activity, functional capacity, disease damage, and therapeutic response in JIA patients from two Brazilian referral centers for pediatric rheumatology, during one-year of a real-life follow-up, and to study predictors for these outcomes on a real-life basis. Characteristics of JIA were also compared according to the different subtypes.

## Methods

This was an observational study of JIA patients followed in two reference centers of pediatric rheumatology in Brazil. The research was carried out at the Pediatric Rheumatology outpatient clinic of the Pediatrics Department of the Universidade Federal de São Paulo, and at the Children’s Institute of Faculdade de Medicina da Universidade de São Paulo. This study was adapted from the INCA study – Inception Cohort of JIA in Latin America. The patients were followed for a period of one-year, recruited between March 2021 and December 2022. The inclusion criteria were: patients aged ≤ 18 years at the beginning of the study, and patients who met the International League of Associations for Rheumatology (ILAR) classification criteria for JIA [11]. Patients who did not have adequate data collection were systematically excluded.

The study was approved by the Ethics and Research Committee of the two University Hospitals. Informed assent and consent were obtained from all JIA patients, and their legal guardians, respectively. The inclusion of the patients occurred at any point of the disease duration. They underwent four visits over a period of 12 months: baseline (Vbaseline), week 12 (V12), week 26 (V26) and week 52 (V52). Demographic, clinical, laboratory, and treatment data were collected during each evaluation. Clinimetric definitions were used to assess disease activity, therapeutic response, disease remission, recurrence, functional capacity, and joint and extra-articular disease damage, at all visits.

The following clinimetric parameters were systematically used:

1. Disease activity according to JADAS 71 [4].
2. Minimal disease activity (MDA) according to Magni-Manzoni [6].
3. Therapeutic response criteria according to JIA-ACR 30/50/70/90 responses [3].
4. Inactive Disease according to Wallace criteria [5].
5. Disease flare, according to definition of disease flare by Brunner [12].
6. Remission on medication according to Wallace criteria [5] at V26 and V52.
7. Remission off medication according to Wallace criteria [5] at V52.
8. Functional capacity using the Childhood Health Assessment Questionnaire (CHAQ) [8].
9. Index of joint and extra-articular damage using the Juvenile Arthritis Damage Index (JADI) [7].
10. Physician Global Assessment (PGA) and patient/caregiver assessment of pain and well-being of the child by VAS.

The following complications were considered as damage related to JIA:

- Osteoporosis; defined by the presence of fracture with vertebral compression or 2 or more fractures of long bones up to 10 years of age, or, 3 or more fractures of long bones at any age up to 19 years associated with low bone mass with Z-score< -2 [13].
- Growth retardation.
- Delay in puberty.
- Local lower limb growth changes.

All data collected during the different visits was stored in Excel spreadsheets.

Statistical analysis was performed using software R 4.2.0. Quantitative variables were described as means and standard deviations (SD), or medians and interquartile range [IQR], according to Kolmogorov-Smirnov normality tests. Qualitative variables were described as absolute numbers (n) and percentages (%). To compare the quantitative variables, either Student t test (comparison between 2 groups), or ANOVA (comparison between more than 2 groups), or mixed model (for measurements taken more than once in the same patient), was used. When the distribution of variables did not follow the normal distribution, either the Wilcoxon-Mann-Whitney test (comparison between 2 groups), or the Kruskal-Wallis test (more than 2 groups compared), was used. For categorical variables, comparisons between the two groups were carried out using either Fisher’s exact test or Chi-square test. Multivariable logistic regression was performed with a stepwise selection to evaluate different baseline variables which are explanatory of different outcomes of interest at V52, such as: disease activity, MDA, disease flare, remission on medication, and remission off medication. All statistical tests required a p-value of ≤ 0.05 to be considered significant.

## Results

A total of 127 JIA patients were included, 113 (89%) completed all four visits, and 83(65.4%) were female. One hundred and eleven (87.4%) patients had been diagnosed before the study initiation, and 16 (12.6%) received JIA diagnosis at the time of study enrollment. Ninety-five (74.8%) patients had prospective evaluation. Median age at disease onset and median elapsed time between first symptom and diagnosis were 4.1 years [2.2; 7.4] and 6.0 months [3.0; 12.0], respectively. Mean age at baseline was 11.0 years [± 4.2], and mean disease duration was 5.8 years [± 4.1]. The demographic and clinical data of the patients and the subtypes of JIA are presented in Table 1.

**Table 1:** Demographic and clinical data of patients with JIA at baseline assessment

Variables	Total (*n* = 127)
Female	83 (65.4)
Age at baseline assessment, years	11.0 ± 4.2
Age at disease onset, years	4.1 [2.2; 7.4]
Age at JIA diagnosis, years	6.2 ± 4.0
Duration of disease, years	5.8 ± 4.1
Elapsed time between first symptom and diagnosis (months)	6.0 [3.0; 12.0]
Subtypes of JIA (%)	
Persistent oligoarticular	39/127 (30.7)
Extended oligoarticular	14/127 (11.0)
RF positive polyarticular	5/127 (3.9)
RF negative polyarticular	39/127 (30.7)
Systemic	16/127 (12.6)
ERA	7/127 (5.5)
Undifferentiated	7/127 (5.5)

JIA- Juvenile Idiopathic Arthritis, RF - rheumatoid factor, ERA – Enthesitis-Related Arthritis

Results of numerical variables represented by median (IQR) or mean ± SD and n (%)

Musculoskeletal involvement and laboratory data, according to the different subtypes of JIA at the baseline assessment, are presented in Table 2 (at the end of the document text file).

**Table 2:** Musculoskeletal involvement and laboratory data in different categories of JIA at baseline

Measure	Persistent oligoarticular (*n* = 39)	Extended oligoarticular(*n* = 14)	Polyarticular RF+ (*n* = 5)	Polyarticular RF- (*n* = 39)	Systemic (*n* = 16)	ERA (*n* = 7)	Undif (*n* = 7)	*p*-value
**Number of Patients**								
Hip	2 (5.1)	0 (0.0)	1 (20.0)	4 (10.2)	4 (25.0)	2 (28.6)	2 (28.6)	0.073
Sacroiliac	0 (0.0)	0 (0.0)	0 (0.0)	0 (0.0)	0 (0.0)	0 (0.0)	1 (14.2)	0.893
Thoracic	0(0.0)	0 (0.0)	0 (0.0)	0 (0.0)	1 (6.2)	0 (0.0)	0 (0.0)	0.322
Active Joints	11 (28.2)	8 (57.1)	3 (60.0)	16 (41.0)	10 (62.5)	2 (28.6)	2 (28.6)	0.182
Limited Joints	14 (35.9)	9 (64.3)	5 (100.0)	23 (59.0)	11 (68.8)	4 (57.1)	4 (57.1)	0.066
Joints with Pain	6 (15.4)	6 (42.9)	1 (20.0)	6 (15.4)	11 (68.8)	2 (28.6)	2 (28.6)	0.001
Increased ESR	8/35 (22.9)	3/12 (25.0)	1 (20.0)	5/34(14.7)	5/13 (38.5)	4 (57.1)	3 (42.9)	0.223
Increased CRP	5/33 (15.2)	1/12 (8.3)	0 (0.0)	6/31 (19.4)	8/13 (61.5)	1 (14.3)	2/6 (33.3)	0.013
Rheumatoid factor +	0 (0.0)	0 (0.0)	5 (100.0)	0 (0.0)	0 (0.0)	0 (0.0)	4/ (57.1)	< 0.001
ANA +	28 (71.8)	9 (64.3)	2 (40.0)	24 (61.5)	4 (25.0)	3 (42.9)	1 (14.3)	0.009
Presence of HLA B27	0/4 (0.0)	0/4 (0.0)	0 (0.0)	0/4 (0.0)	0/5 (0.0)	2 (28.6)	1/4 (25.0)	0.480
ESR	10.0 [5.5; 17.5] (*n* = 35)	10.0 [4.8; 22.5] (*n* = 12)	6.0 [5.0; 4.0]	9.0 [5.0; 14.2] (*n* = 34)	8.0 [5.0; 56.0] (*n* = 13)	33.0 [9.5; 38.0]	7.0 [5.5; 32.0]	0.694
**Number of Joints**								
Active joints	0.0 [0.0; 1.0]	1.0 [0.0; 4.0]	3.0 [0.0; 5.0]	0.0 [0.0; 4.0]	1.5 [0.0; 6.2]	0.0 [0.0; 1.0]	0.0 [0,0; 0,5]	0.030
Limited joints	0.0 [0.0; 1.0]	3.0 [0.0; 3.8]	3.0 [20; 6.0]	2.0 [0.0; 6.0]	2.5 [0.0; 6.0]	1.0 [0.0; 1.5]	1.0 [0.0; 2.5]	0.001
Joint pain	0.0 [0.0; 0.0]	0.0 [0.0; 1.0]	0.0 [0.0; 0.0]	0.0 [0.0; 0.0]	2.0 [0.0; 4.5]	0.0 [0.0; 1.0]	0.0 [0.0; 0.5]	0.001

ERA: Enthesitis Related Arthritis, Undif: undifferentiated, ESR: erythrocyte sedimentation rate, CRP: C-reactive protein, ANA: antinuclear antibody, HLA B27: human leukocyte antigen B27

Results in median (IQR) and n (%)

At the baseline, we observed a more significant number of patients with joint pain (*p* = 0.001), especially those with systemic JIA, as well as a significant decrease in the number of patients with elevated CRP over the time, with differences between the groups (systemic JIA vs. extended oligoarticular JIA, *p* = 0.011; systemic JIA vs. persistent oligoarticular JIA, *p* = 0.003; and systemic JIA vs. RF-negative polyarticular JIA, *p* = 0.012).

JADAS71, parents’/patients’ VAS of pain, parents’/patients’ VAS of overall well-being, PGA, and CHAQ improved during the follow-up (*p* < 0.05). MDA was present in 80.6% at V52 (*p* < 0.035 among the visits). The number of patients with inactive disease or MDA increased significantly over time, as did the number of patients fulfilling JIA-ACR 30, 50, 70, and 90 assessments (*p* < 0.05). The number of patients with positive CHAQ decreased over time (*p* < 0.05) (Table 3) (at the end of the document text file).

**Table 3:** Clinimetric data of JIA patients during the four visits (V) of the study

Variables	V baseline	V12	V26	V52	*p*-value
JADAS 71, mean ± SD	7.05 ± 9.3 (*n* = 119)	4.24 ± 7.1 (*n* = 126)	3.59 ± 7.2 (*n* = 124)	3.52 ± 8.4 (*n* = 110)	< 0.001
Parents/patients’ VAS of pain, mean ± SD	1.75 ± 2.3 (*n* = 122)	1.25 ± 2.1 (*n* = 126)	1.15 ± 2.1 (*n* = 123)	1.0 ± 2.2 (*n* = 111)	< 0.001
Parents/patients’ VAS overall well-being, mean ± SD	2.49 ± 2.7 (*n* = 122)	1.17 ± 1.7 (*n* = 126)	1.10 ± 2.0 (*n* = 122)	0.85 ± 1.7 (*n* = 111)	< 0.001
PGA, measured by VAS, mean ± SD	2.50 ± 2.8 (*n* = 122)	1.72 ± 2.6 (*n* = 126)	1.29 ± 2.2 (*n* = 122)	1.06 ± 2.3 (*n* = 111)	< 0.001
CHAQ, mean ± SD	0 ± 0.5 (*n* = 114)	0.21 ± 0.4 (*n* = 120)	0.19 ± 0.5 (*n* = 112)	0.14 ± 0.4 (*n* = 101)	0.018
JADI-A, mean ± SD	1.15 ± 3.4 (*n* = 122)	0.87 ± 2.9 (*n* = 126)	1.08 ± 3.4 (*n* = 126)	1.22 ± 3.8 (*n* = 111)	0.407
JADI-E, mean ± SD	0.33 ± 0.7 (*n* = 127)	0.42 ± 1.2 (*n* = 127)	0.33 ± 0.7 (*n* = 127)	0.36 ± 0.9 (*n* = 113)	0.366
**Number of Patients**					
CHAQ amended > 0 n (%)	45/114 (39.5)	41/120 (34.2)	26/112 (23.2)	20/101 (19.8)	< 0.001
Inactive disease, n (%)	41/123 (33.3)	60/126 (47.6)	82/124 (66.1)	74/109 (67.9)	< 0.001
MDA, n (%)	64/109 (58.7)	80/111 (72.0)	85/108(78.7)	79/98(80.6)	0.035
Disease flare, n (%)	12/120 (10.0)	9/124 (7.3)	7/122 (5.7)	8/109 (7.3)	0.635
Remission on medication n (%)			52/127 (40.9)	52/113 (46.0)	
Remission off medication n (%)				12/113 (10.6)	
JIA-ACR 30, n (%)	95/119 (79.8)	105/122 (86.1)	108/119 (90.8)	95/107 (88.8)	0.027
JIA-ACR 50, n (%)	85/119 (71.4)	99/122 (81.1)	105/119 (88.2)	90/107 (84.1)	0.003
JIA-ACR 70, n (%)	71/119 (59.7)	83/122 (68.0)	91/119 (76.5)	83/107 (77.6)	0.010
JIA-ACR 90, n (%)	59/119 (49.6)	74/122 (60.7)	80/119 (67.2)	74/107 (69.2)	0.003

JADAS: Juvenile Arthritis Disease Activity Score, V: visit, VAS: Visual Analogue Scale, PGA: Physician Global Assessment, CHAQ: Childhood Health Assessment Questionnaire, JADI-A: Juvenile Arthritis Damage Index-Joint, JADI-E: JADI-Extra-articular, MDA: Minimal Disease Activity, JIA-ACR: Therapeutic Response Criteria of the Pediatric ACR

The baseline variables associated with disease activity, MDA, disease remission, disease flare, and remission on medication, at V52 were assessed. The following factors shown in Table 4at baseline were associated with disease activity at V52: presence of active joints (*p* = 0.009) and presence of limited joints (*p* = 0.002). For each additional number of joints with pain, the chance of being in disease activity at V52 was 0.34 greater than Vbaseline (OR = 1.34; CI 95% 1.15 to 1.63, *p* = 0.001).

**Table 4:** Association between disease activity at visit 52 and categorical variables at baseline

Variables	Categories	Disease activity	*p*-value
**Total**		**0.0 [0.0; 2.8] (** ***n*** ** = 110)**	
Active Joints	Not	0.0 [0.0; 1.0] (*n* = 65)	0.009
	Yes	0.0 [0.0; 7.0] (*n* = 45)	
Limited Joints	Not	0.0 [0.0; 0.0] (*n* = 48)	0.002
	Yes	0.0 [0.0; 6.5] (*n* = 62)	
Joints with Pain	Not	0.0 [0.0; 1.5] (*n* = 79)	0.051
	Yes	0.6 [0.0; 6.5] (*n* = 31)	

The presence of active and limited joints, the number of active joints, the number of joints with pain, and the limited number of joints at Vbaseline were associated with MDA at V52 (Table 5). One additional joint with pain at Vbaseline decreased the chance of achieving MDA at V52 (OR = 0.79; CI 95% 0.65 to 0.96, *p* = 0.018) by 21%.

**Table 5:** Association between MDA at visit 52 and categorical variables at baseline

Variables	MDA		*p*-value
	Not (*n* = 19)	Yes (*n* = 79)	
Active Joints	11 (57.8%)	26 (32.9%)	0.027
N° of Active joints	2.0 [0.0; 11.0]	0.0 [0.0; 1.0]	0.004
Limited Joints	15(78.9%)	39 (49.3%)	0.003
N° of Limited Joints	3.0 [2.0; 6.0]	1.0 [0.0; 2.0]	< 0.001
Joints with Pain	6 (31.5%)	15 (18.9%)	0.098
N° of Joints with Pain	0.0 [0.0; 6.0]	0.0 [0.0; 0.0]	0.015

N°: Number, MDA: Minimal Disease Activity

None of the variables were associated with the presence of flare or with the presence of remission on medication at V52. Table 6 shows the association between remission off medication at V52 and baseline variables. Only the presence and number of active joints were associated with remission off medication. The presence of active joints at baseline was associated with a lower remission rate at V52, with an 84% reduction in the chance of the patient going into remission off medication (OR = 0.16; CI 95% from 0.03 to 0.91, *p* = 0.039). Disease duration was assessed, but no association with the remission rate (*p* = 0.644) was found. There were no differences in relation to disease activity at the last visit among the different subtypes of JIA.

**Table 6:** Association between remission off medication at visit 52 and variables at baseline

Variables	Remission off medication		*p*-value
	No (*n* = 101)	Yes (*n* = 12)	
Active Joints	46/101 (45.5%)	1/12 (8.3%)	0.014
N° of Active joints	0.0 [0.0; 3.0]	0.0 [0.0; 0.0]	0.012
Limited Joints	60/101 (59.4%)	5/12 (41.7%)	0.355
N° of Limited Joints	1.0 [0.0; 4.0]	0.0 [0.0; 2.0]	0.158
Joints with Pain	31/101 (30.7%)	1/12 (8.3%)	0.174
N° of Joints with Pain	0.0 [0.0; 1.0]	0.0 [0.0; 0.0]	0.085

N°: Number

Musculoskeletal involvement and laboratory data of all JIA patients during the study visits are illustrated in Table 7 (at the end of the document text file). There was a significant decrease in the number of JIA patients with active, limited, and painful joints (*p* < 0.001, *p* < 0.001, *p* < 0.002, respectively). There was also a significant decrease in the number of active joints and limited joints over the follow-up time (*p* = 0.001 and *p* = 0.002). Significant decrease in the number of JIA patients with increased ESR (> 20 mm/1st hour) and increased CRP were observed over the follow-up time (*p* = 0.001 and *p* < 0.001, respectively), as well as significant reduction in ESR value at the 26 V compared to the 12 V (*p* = 0.006).

**Table 7:** Musculoskeletal involvement and laboratory data of JIA patients during the four visits (V) of the study

Variables	V baseline *N* = 127	V12 *N* = 127	V26 *N* = 127	V52 *N* = 113	*p*-value
**N**°** of patients**					
Hip involvement (%)	15 (11.8)	9 (7)	9 (7.1)	11 (9.7)	0.126
Sacroiliac involvement (%)	0 (0)	0 (0)	0 (0)	1 (0.9)	> 0.999
Thoracic involvement (%)	1 (0.8)	1 (0.8)	0 (0)	0 (0)	> 0.999
Active joints (%)	52 (40.9)	40 (31.5)	29 (22.8)	21 (18.6)	< 0.001
Limited joints (%)	70 (55.1)	67 (52.8)	57 (44.9)	49 (43.4)	< 0.001
Joints with pain (%)	34 (26.8)	28 (22.0)	20 (15.7)	16 (14.2)	0.002
ESR increase (%)	29/113 (25.7)	31/111 (27.9)	14/97 (14.4)	16/95 (16.8)	0.001
CRP increase (%)	23/106 (21.7)	17/96 (17.7)	11/94 (11.7)	11/93 (11.8)	< 0.001
Rheumatoid factor + (%)	9 (7.1)	-	-	-	-
ANA + (%)	71 (55.9)	-	-	-	-
HLA B27 + (%)	3/29 (10.3)	-	-	-	-
ESR value	10.0 [5.0; 21.0] (*n* = 113)	8.0 [5.0; 24.5] (*n* = 111)	10.0 [5.0;16.0] (*n* = 97)	10.0 [5.0;17.0] (*n* = 95)	0.387
**Number of joints**					
Active joints	2.37(± 5.2)	1.25 (± 3.4)	1.19(± 3.7)	1.35(± 4.1)	< 0.001
Limited joints	2.97 (± 5.4)	2.56 (± 5.47)	2.40 (± 5.7)	2.07 (± 5.17)	0.002
Joint pain	1.40 (± 4.2)	0.87 (± 3.4)	0.92 (± 3.7)	1.05 (± 3.8)	0.186

ESR: erythrocyte sedimentation rate, CRP: C-reactive protein, ANA: Antinuclear Antibody, HLA B27: human leukocyte antigen B27, V: visit. Results in median (IQR) or mean ± SD and n (%)

Enthesitis was observed in 4 (3.1%) patients at baseline, with improvement during follow-up, occurring in one (0.78%) patient at V52. Active uveitis occurred in 17 (13.4%) patients: 10 (7.9%) at baseline, 7 (5.5%) at V12, 4 (3.1%) at V26, and 3 (2.7%) at V52, with significant improvement during follow-up (*p* = 0.047). The most frequent subtype of JIA in patients with uveitis was persistent oligoarticular JIA (8 [6.3%]), followed by ERA JIA (4 [3.1%]), RF-negative polyarticular JIA (3 [2.4%]), RF positive polyarticular JIA (1 [0.8%]) and undifferentiated JIA (1 [0.8%]).

Growth retardation was observed in 9 (7.1%) patients at baseline, 11 (8.7%) at V12, 6 (4.7%) at V26, and 6 (4.7%) at V52. 2/112 (1.8%) patients had delay in puberty at baseline and at all visits, and 4 (3.1%) had localized lower limb growth changes that continued over time. Osteoporosis was diagnosed in 2 (1.6%) patients with no statistical difference between the evaluations.

Regarding treatment at baseline, 12% of patients were on systemic corticosteroids, 11.9% were on non-steroidal anti-inflammatory drugs (NSAIDs), 84.9% were on synthetic disease-modifying antirheumatic drugs (sDMARDs), and 51.2% were on biological DMARDs (bDMARDs). There was a significant decrease in the use of sDMARDs between V26 and V52 (*p* = 0.006). The most used sDMARDs at baseline visit was methotrexate (used by 61.1% of the patients), followed by leflunomide (20.6%), cyclosporine (6.3%) and sulfasalazine (0.8%). Regarding bDMARDs, there was no difference in their use over time. Adalimumab was the most used bDMARD (used by 27.1% of the patients) at baseline visit, followed by tocilizumab (13.0%), etanercept (10.6%), and infliximab (1.6%).

## Discussion

This study assessed clinical, laboratory, and outcome data, of patients with JIA in two Brazilian referral centers in Pediatric Rheumatology during 12 months of follow-up. Some aspects of this study, such as scarcity of JIA studies in Latin America, assessment of the applicability of JIA treatment protocols in a country with low resources, clinimetric measurements over one year of follow-up in real life, and the assessment of MDA, a relatively new concept in JIA [6, 14], give it great relevance.

Over the course of the four evaluations, we found improvements in the following clinical parameters: number of patients with active, painful and limited joints, disease activity measured by JADAS 71, VAS of the child pain for parents and patients, VAS of the child well-being for parents and patients, and PGA. We also observed improvements in the total number of active and limited joints. Laboratory parameters, such as CRP and ESR levels, and the therapeutic response criteria, measured by ACR Ped 30, 50, 70 and 90, also showed progressive improvement. The percentages of patients with MDA and patients with inactive disease also increased during the follow-up, achieving 80.6% and 67.9%, respectively, at last evaluation. Clinimetric measurements, although relevant for follow-up and monitoring of therapeutic response, have rarely been used in the pediatric literature, especially in developing countries. In our sample, we observed an improvement in disease activity status, measured by JADAS71, over 12 months of follow-up. At final assessment, approximately half of patients were in remission on medication, about 10% in remission off medication, and more than two-thirds were inactive. The literature also shows favorable outcomes in contrast to the pre-biological era. In a multicenter study from North America with 2904 patients with JIA, a high proportion of patients with inactive disease was observed after one year of follow-up, measured by the clinical JADAS10 (cJADAS10), parents’/patients’ VAS, and PGA [15]. Castillo-Vilella et al. observed that 70% of the patients in their cohort achieved clinical remission, and 29% remission off medication, in an observation period of 2 years in a prospective cohort, and one year in a retrospective cohort [16]. In another study, the authors observed that 47% of patients were in remission off medication, 25% in remission on medication, 27% with active disease, and 51% were using at least one DMARD (of these, 22% were biological), after 3 years of follow-up [2]. A recent review included JIA studies with patients that were followed for a minimum period of 2 years, and revealed that almost half of them were in disease remission at the final assessment [17].

It is worth noting that the regression analysis revealed some predictors for outcomes at final assessment. The presence of active joints at baseline reduced the chance of the patient going into remission off medication, and the presence of each additional unit in the number of joints with pain at the baseline, decreased the chance of achieving MDA. Predictors of severity and remission have been studied in JIA, and some variables such as high disease activity, involvement of ankles, wrists, and hips, and delay in the beginning of treatment seemed to predict long-term disease severity [18].

Our study revealed a significant increase in patients with MDA over the course of the follow-up. A cross-sectional study showed MDA in 44.8 to 62.4%, and in 44.6 to 50.4% of two cohorts of patients with oligoarticular JIA and polyarticular JIA, respectively [19]. In our cohort, we observed MDA in 80.6% at the final visit. To the best of our knowledge, there are no longitudinal studies in the literature regarding MDA.

Although most patients were not included at the beginning of their diagnosis and treatment, follow-up revealed a favorable evolution. Regarding the patients included soon after diagnosis, we observed that the clinical characteristics and the classification of subtypes were not statistically different.

Confirming a favorable follow-up, we observed that although about half of the patients presented joint limitations throughout, these were restricted to a small number of joints (with a maximum mean of two joints). The functional capacity of these patients, as measured by CHAQ, also showed improvement during follow-up.

We observed that the number of patients using sDMARDs decreased over time, especially the use of methotrexate. On the other hand, the number of patients using bDMARDs did not present significant differences over time, which is in accordance with what we are used to doing in our clinical practice (tapering the sDMARDs first). Therefore, a probable explanation for the reduction in the use of sDMARDs was the clinical improvement presented by the patients during follow-up. Regarding corticosteroid dose, we observed that few patients continued the medication, with no significant decrease in dose over time. This was due to the presence of disease flare in some patients with the need for increase of, or resumption of, this medication.

This study revealed a relatively short time (6 months) to JIA diagnosis, which surprised us, positively, since it shows greater awareness about the disease on the part of physicians, with subsequent earlier referral to a specialist, compared to the past. A study conducted in the city of Sao Paulo in 2002 showed a mean delay to diagnosis of 1.4 years, which had a range between a few days and 10 years, and a mean of non-specialist physician consultations before visiting a specialist, of 3.6 years [20]. The favorable outcome of the patients in our study could be explained in part by the earlier treatment and is also demonstrated by the low frequency of comorbidities. However, the subtypes with polyarticular involvement presented more joint limitation.

This study has strengths, particularly the use of concomitant validated clinimetric tools to evaluate disease activity, disease remission, functional capacity, damage, and therapeutic response. Indeed, these instruments are relevant in assessing the overall JIA during follow-up. The main limitation of our study is that most patients were included at different stages of the disease, with only a small proportion (12.6%) included at the time of diagnosis, which makes adequate comparisons with other cohorts difficult.

## Conclusion

This real-life study with JIA patients conducted over one-year of follow-up revealed improvement of the disease across several parameters, measured through validated scores of disease activity, functional capacity, therapeutic response, and disease damage. An association between the presence of active joints at baseline and reduced chance of achieving remission after one year was observed. However, more than half of the patients were in remission of the disease after this time. Long-term prospective and multicenter studies are needed to better assess the outcome of JIA and the differences among JIA subtypes.

## Data Availability

All data generated during this study is available from the corresponding author on reasonable request.

## Abbreviations

ACR: American College of Rheumatology
ANA +: Antinuclear antibody
bDMARDs: Biological disease-modifying antirheumatic drugs
CHAQ: Childhood Health Assessment Questionnaire
CRP: C-reactive protein
ERA: Enthesitis Related Arthritis
ESR: Erythrocyte sedimentation rate
HLA B27: Human leukocyte antigen B27
ILAR: International League of Associations for Rheumatology (ILAR)
JADAS: Juvenile Arthritis Disease Activity Score
JADI: Juvenile Arthritis Damage Index
JADI-A: Juvenile Arthritis Damage Index-Joint Damage
JADI-E: Juvenile Arthritis Damage Index-Extra-articular Damage
JIA: Juvenile idiopathic arthritis
JIA-ACR: Therapeutic Response Criteria of the Pediatric American College of Rheumatology
MDA: Minimal Disease Activity
N°: Number
NSAIDs: Non-steroidal anti-inflammatory drugs
RF: Rheumatoid fator
sDMARDs: Synthetic disease-modifying antirheumatic drugs
PGA: Physician Global Assessment
Undif: Undifferentiated
V: Visit
